# Identification and profiling of *Cyprinus carpio* microRNAs during ovary differentiation by deep sequencing

**DOI:** 10.1186/s12864-017-3701-y

**Published:** 2017-04-28

**Authors:** Fang Wang, Yongfang Jia, Po Wang, Qianwen Yang, QiYan Du, ZhongJie Chang

**Affiliations:** College of Life Science, Henan Normal University, Xinxiang, Henan 453007 People’s Republic of China

**Keywords:** miRNA, Deep sequencing, Ovary development, *Cyprinus carpio*

## Abstract

**Background:**

MicroRNAs (miRNAs) are endogenous small non-coding RNAs that regulate gene expression by targeting specific mRNAs. However, the possible role of miRNAs in the ovary differentiation and development of fish is not well understood. In this study, we examined the expression profiles and differential expression of miRNAs during three key stages of ovarian development and different developmental stages in common carp *Cyprinus carpio*.

**Results:**

A total of 8765 miRNAs were identified, including 2155 conserved miRNAs highly conserved among various species, 145 miRNAs registered in miRBase for common carp, and 6505 novel miRNAs identified in common carp for the first time. Comparison of miRNA expression profiles among the five libraries identified 714 co-expressed and 2382 specific expressed miRNAs. Overall, 150, 628, and 431 specifically expressed miRNAs were identified in primordial gonad, juvenile ovary, and adult ovary, respectively. MiR-6758-3p, miR-3050-5p, and miR-2985-3p were highly expressed in primordial gonad, miR-3544-5p, miR-6877-3p, and miR-9086-5p were highly expressed in juvenile ovary, and miR-154-3p, miR-5307-5p, and miR-3958-3p were highly expressed in adult ovary. Predicted target genes of specific miRNAs in primordial gonad were involved in many reproductive biology signaling pathways, including transforming growth factor-β, Wnt, oocyte meiosis, mitogen-activated protein kinase, Notch, p53, and gonadotropin-releasing hormone pathways. Target-gene prediction revealed upward trends in miRNAs targeting male-bias genes, including *dmrt1*, *atm*, *gsdf*, and *sox9*, and downward trends in miRNAs targeting female-bias genes including *foxl2*, *smad3*, and *smad4*. Other sex-related genes such as *sf1* were also predicted to be miRNA target genes.

**Conclusions:**

This comprehensive miRNA transcriptome analysis demonstrated differential expression profiles of miRNAs during ovary development in common carp. These results could facilitate future exploitation of the sex-regulatory roles and mechanisms of miRNAs, especially in primordial gonads, while the specifically expressed miRNAs represent candidates for studying the mechanisms of ovary determination in Yellow River carp.

**Electronic supplementary material:**

The online version of this article (doi:10.1186/s12864-017-3701-y) contains supplementary material, which is available to authorized users.

## Background

MicroRNAs (miRNAs) are single-stranded, highly-conserved, non-coding RNA molecules of 19–24 nucleotides (nt), which regulate gene expression by targeting specific sites in the 3’ untranslated region of mRNAs at the post-transcriptional level [1–3]. MiRNAs induce repression of mRNA translation or transcript destabilization, and have thus been shown to play important roles in controlling multiple biological processes such as embryonic development, cell cycle control, apoptosis, cell proliferation and differentiation, and immune and stress responses in various organs [4–8]. Genome-wide miRNAs have also been shown to play a vital role in gonad development in mammals including mice [7, 9], pigs [10], cattle [11], and sheep [12]. Seven miRNAs (bta-miR-143, bta-let-7f, bta-let-7a, bta-let-7c, bta-miR-10b, bta-let-7b, and bta-miR-26a), each with >100,000 reads, were the most abundant in Holstein cattle [11], while miR-224 was involved in the regulation of follicular development in mice [13]. Some miRNAs have been reported to regulate proliferation and apoptosis, as well as estradiol production, in porcine and mouse granulosa cells, suggesting that miRNAs also play a critical role in regulating follicle development and oocyte maturation in mammals [14–17]. However, studies on the functions of miRNAs in sex development and differentiation are limited, especially in fish. mRNA and miRNA expression have been investigated in gonads, as the primary organs of sexual reproduction [18], and several studies have revealed divergent miRNA expression in testes and ovaries of various animals, suggesting an important role for miRNAs in driving sex differentiation and development [9, 19, 20]. MiR-200b expression levels in the flounder *Paralichthys olivaceus* were seven-fold higher in the ovary compared with the testis, indicating female-biased miRNA expression, while Pol-miR-726 was identified as a unique miRNA in *P. olivaceus* ovary [21]. In yellow catfish, 23, 30, and 14 miRNAs were specifically detected in XX ovary, XY testis, and YY testis, respectively [22]. Testicular expression of miR-2184 in medaka was significantly higher than in all other tissues [23]. Given that the primordial gonad represents the initial stage of gonadal development and a critical period of sex determination and differentiation, it is important to understand the types and characteristics of the miRNAs involved during this period.

The common carp, *Cyprinus carpio*, is one of the most important cyprinid species and accounts for 10% of global freshwater aquaculture production [24]. Genomic studies of common carp have recently made extensive progress. Its transcriptome was deep-sequenced by Ji et al. [25], and Jian et al. [26] identified changes at the transcriptomic level in common carp spleen following 24-h experimental infection with Aeromonas hydrophila. A large number of gene-associated single-nucleotide polymorphisms were identified in four strains of common carp using next-generation sequencing [27]. MiRNAs and miRNA-related SNPs were also identified, and miRNA-related single-nucleotide polymorphisms have been shown to affect miRNA biogenesis and regulation in the common carp [28]. Yellow River carp refers to common carp from the Yellow River, which are famous in China for their tender, tasty, and nutritional meat. Females grow faster than males, which makes the mechanism of sex differentiation and development an intriguing topic in this commercial species [29, 30]. However, there is relatively little information about Yellow River carp, especially in terms of the stage-specific miRNA characteristics of the ovary. Identifying and characterizing the temporospatial characteristics of miRNAs is thus the first step in elucidating the roles of miRNAs in gonadal development and differentiation in this fish.

It would be valuable to understand the relative expression and roles of miRNAs during key stages of ovarian development, including primordial gonad, juvenile ovary, and adult ovary. Furthermore, the neurula stage is the starting point for nervous system development, and the hypothalamic–pituitary–gonad (HPG) axis plays an important role in the sex differentiation of fish [31]. Environmental factors also affect sex differentiation in fish, and even play a decisive role in some species, and the complete yolk sac absorption stage represents the point at which environmental impacts start to have an effect [32, 33]. It is therefore necessary to analyze the types and characteristics of miRNAs in the neurula and yolk sac absorption stages to provide a comprehensive understanding of the role of miRNAs in ovary differentiation.

In this study, we therefore aimed to construct *C. carpio* miRNA libraries from primordial gonad, juvenile ovary, and adult ovary, and from neurula and yolk sac absorption stage embryos to investigate changes in miRNA expression profiles during ovarian differentiation. We constructed five small-RNA libraries from five different developmental stages of Yellow River carp to identify differentially expressed and novel miRNAs, which may play regulatory roles in ovary differentiation. The results of this study may provide the basis for a better understanding of the roles of miRNAs in ovarian differentiation, leading to an ability to exploit the mechanisms of sexual regulation in Yellow River carp.

## Results

### Construction of cDNA libraries for sequencing and identification of miRNAs

We constructed cDNA libraries of small RNAs using pooled total RNAs from primordial gonad, juvenile ovary, and adult ovary, and neurula and complete yolk sac absorption stage embryos, respectively. Totals of 16,968,093, 15,722,656, 14,604,487, 16,247,107, and 15,228,538 reads, respectively, were obtained from the five libraries using Solexa sequencing (Additional file 1). After filtering out low-quality sequences, 5’ and 3’ adapters, and reads <18 nt, 15,765,913, 15,157,370, 13,750,335, 15,122,610, and 14,250,458 clean reads were obtained for further analysis (Table 1). After comparing the small RNA sequences with NCBI GenBank and RFam, known types of RNA sequences including rRNA (6.93, 14.07, 3.12, 0.87, and 3.34%, respectively), small nuclear RNA (snRNA), small nucleolar RNA (snoRNA), and tRNA (0.24, 0.19, 0.04, 0.01, and 0.05%, respectively), and repeat sequences were removed.

**Table 1:** Distribution of sequenced reads from raw data to cleaned sequences

Type	neurula stage		yolk sac complete absorption stage		primordial germ cells stage		juvenile ovary		adult ovary	
	Total reads	%	Total reads	%	Total reads	%	Total reads	%	Total reads	%
Raw reads	16247107	100	15228538	100	1696809	100	15722656	100	14604487	100
tRNA	4333	0.24	1	0.19	1037	0.04	465	0.01	2137	0.05
snoRNA	54	0.003	77	0.012	72	0.003	92	0.001	49	0.001
rRNA	127709	6.93	87777	14.07	87728	3.12	63655	0.87	148915	3.34
snRNA	1	0	1	0	0	0	3	0	2	0
Other	1709552	92.83	534674	85.72	2722236	96.84	7213966	99.12	4301005	96.61

**Fig. 1:**
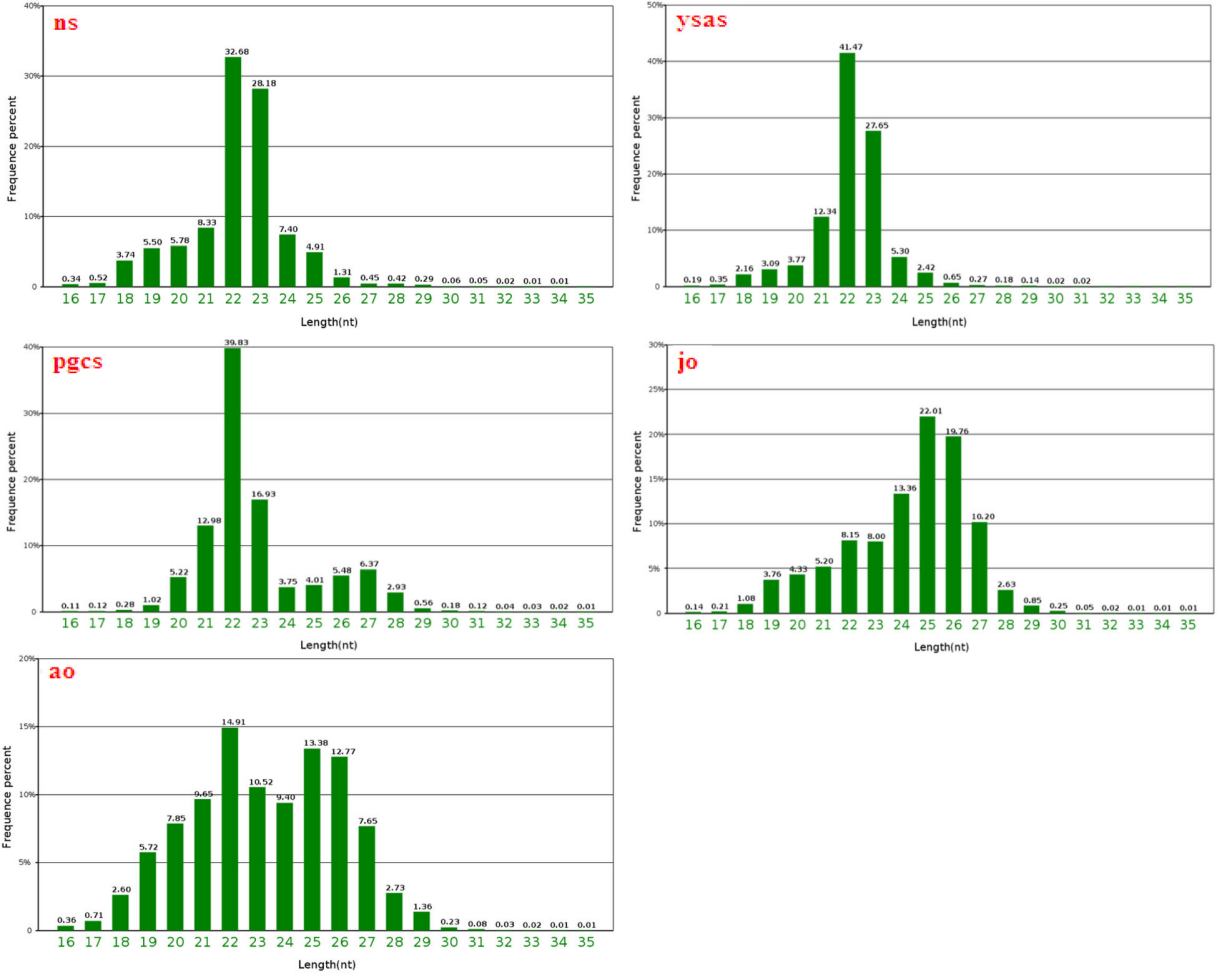
Length distribution of miRNA sequences in neurula stage (ns), complete yolk sac absorption stage (ysas), primordial gonads (pgcs), juvenile ovary (jo), and adult ovary (ao) from Yellow River carp by Illumina small-RNA deep sequencing

The length distributions of the high-quality reads varied among the five libraries. The length distributions were similar among the neurula stage, yolk sac absorption stage, and primordial gonad, with 22- or 23-nt small RNAs accounting for 60.86, 69.12, and 56.76% of the total sequences, respectively (Fig. 1). The size distribution increased in juvenile and adult ovary libraries. The length distribution in the adult ovary library was 16–30 nt, with peaks at 22 and at 25–26 nt, which accounted for 41.06% of reads. MiRNAs in the juvenile ovary library ranged from 18 to 29 nt, with a peak at 25–26 nt accounting for 41.77% of reads. These miRNAs corresponded to Piwi-interacting RNAs (piRNAs) (Fig. 1), which are endogenous small non-coding RNA molecules ranging from 26 to 31 nt in length. Various studies have demonstrated that Piwi–piRNA complexes are essential in gene silencing and transposon regulation during germ cell differentiation and gonadal development in animals [34–36].

### Characterization of miRNAs at different ovary development stages in Yellow River carp

To identify conserved miRNAs and predict novel miRNAs in the five libraries, we compared the mapped sequences against reference genomes and aligned them with known mature miRNAs in miRBase 21.0, allowing no more than two mismatches [37]. Totals of 517, 946, 942, 989 and 737 conserved miRNAs were identified from the five libraries (primordial gonad, juvenile ovary, and adult ovary, and neurula and complete yolk sac absorption stage embryos) respectively, which were highly conserved in other species (Additional file 2). These conserved miRNAs had a broad range of expression levels in carp, ranging from 3,683,785 counts for the most abundant to a single count. Among them, miR-430-3p, miR-19-3p, miR-143-3p, miR-202-5p, miR-30-5p, miR-122-5p, let-7-5p, miR-451-5p, miR-223-3p, and miR-216-5p were the highest-expressed miRNAs in the five periods. Existing carp miRNAs were registered in miRBase of common carp, including 145, 139, 143, 143, and 145 existing carp miRNAs (i.e., miRNAs included in *C. carpio* miRBase) from the five libraries, respectively, of which miR-430, miR-21, let-7a, miR-199-5p, miR-9-3p, miR-22a, miR-125b, miR-26a, miR-181a, miR-101a, miR-143, and miR-200a were the most abundant (Additional file 3). To get a clearer perspective of the most abundant conserved miRNAs and existing carp miRNAs, we compared the miRNAs with the 10 highest read numbers (Tables 2 and 3). MiR-430-3p and miR-30-5p were the dominantly expressed conserved miRNAs in primordial germ cells, with >100,000 reads (Table 2), while ccr-miR-21 and ccr-let-7a were the dominantly expressed existing carp miRNAs in primordial gonads, with >10,000 reads (Table 3). Generally, expression levels of existing carp miRNAs were higher than of conserved miRNAs (Tables 2 and 3).

**Table 2:** Ten abundant conserved miRNAs identified in Yellow River carp

miRNA	Sequence	neurula stage	yolk sac complete absorption stage	primordial germ cells stage	juvenile ovary	adult ovary
		Frequency				
miR-430-y	AAAGTGCTATCAAGTTGGGGTA	3674885	8777	55	59	9
miR-19-y	TGTGCAAATCTATGCAAAACTGA	2944128	238631	65965	9627	12050
miR-143-y	TGAGATGAAGCACTGTAGCTCT	989	37873	352148	17495	53756
miR-202-x	TTCCTATGCATATACCTCTTTGA	684	63	147077	35682	271282
miR-30-x	TGTAAACATCCTTGACTGGA	13046	104211	293179	10000	12482
miR-122-x	TGGAGTGTGACAATGGTGTTTG	256	87622	170427	51666	768
let-7-x	TGAGGTAGTAGATTGAATAGTT	600	41641	180455	21923	57391
miR-451-x	AAACCGTTACCATTACTGAGTT	447	2470	199868	13562	8756
miR-223-y	TGTCAGTTTGTCAAATACCCCA	299	9681	165179	8226	20688
miR-216-x	TAATCTCAGCTGGCAACTGTGA	982	64822	92757	4452	275

**Table 3:** Ten abundant existing carp miRNAs identified in Yellow River carp

miRNA	Sequence	neurula stage	yolk sac complete absorption stage	primordial germ cells stage	juvenile ovary	adult ovary
		Frequency				
ccr-miR-430	TAAGTGCTATTTGTTGGGGTAG	1846122	10701	11	15	4
ccr-miR-21	TAGCTTATCAGACTGGTGTTGGC	128047	191505	772028	42406	158595
ccr-let-7a	TGAGGTAGTAGGTTGTATAGTT	1455	50461	407120	94104	328220
ccr-miR-199-5p	CCCAGTGTTCAGACTACCTGTTC	1656	511142	366414	13362	15179
ccr-miR-9-3p	TCTTTGGTTATCTAGCTGTATG	5022	852887	1529	1272	405
ccr-miR-22a	AAGCTGCCAGCTGAAGAACTGT	28851	202470	400577	39619	136304
ccr-miR-125b	TCCCTGAGACCCTAACTTGTGA	839	255585	270975	37470	47113
ccr-miR-26a	TTCAAGTAATCCAGGATAGGCT	18731	144765	360828	25777	50053
ccr-miR-181a	AACATTCAACGCTGTCGGTGA	2652	413308	144273	7212	14370
ccr-miR-101a	TACAGTACTGTGATAACTGAAG	6654	58068	389930	14286	40151

We also identified 6505 miRNAs not previously found in Yellow River carp, including 3,278, 3,257, 3,188, 4,480 and 3,192 in each developmental period (primordial gonad, juvenile ovary, and adult ovary, and neurula and complete yolk sac absorption stage embryos), respectively (Additional file 4). These miRNAs were considered as Yellow River carp-specific candidate miRNAs, and require further investigation. Eleven miRNAs (novel-m4414-5p, novel-m0352-3p, novel-m3425-3p, novel-m1635-5p, novel-m0568-5p, novel-m3309-5p, novel-m3308-5p, novel-m0297-5p, novel-m2780-3p, novel-m0636-5p, novel-m4313-3p), each with >1,000 reads, were the most abundant. We further investigated the most-abundant novel miRNAs in each period by comparing the 11 miRNAs with the highest read numbers (Table 4). Novel-m0352-3p and novel-m3425-3p were the dominantly expressed novel miRNAs in primordial gonads, with 2,102 reads (Table 4). Some novel miRNAs were developmental-stage specific, suggesting that they may perform stage-specific functions. However, further studies are needed to confirm their roles in development.

**Table 4:** Ten abundant novel miRNAs identified in Yellow River carp

miRNA	Sequence	neurula stage	yolk sac complete absorption stage	primordial germ cells stage	juvenile ovary	adult ovary
		Frequency				
novel-m4414-5p		4669	111	22	9986	3590
novel-m0352-3p		598	2534	2102	5	22
novel-m3425-3p		598	2534	2102	5	22
novel-m1635-5p		418	195	122	290	288
novel-m0568-5p		418	195	122	289	288
novel-m3309-5p		159	838	282	20	5
novel-m3308-5p		159	838	282	20	5
novel-m0297-5p		886	176	199	9	2
novel-m2780-3p		2	1223	9	0	1
novel-m0636-5p		825	268	38	0	9

**Fig. 2:**
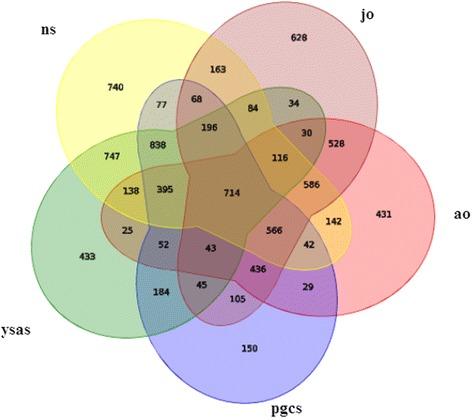
Venn diagram comparing the expression distribution of all the expressed miRNAs in Yellow River carp. Numbers in parentheses represents numbers of co-expressed or differentially expressed miRNAs

A total of 714 miRNAs (29.97%) including 143 existing carp miRNAs, 254 conserved miRNAs and 317 novel miRNAs were co-expressed in all five libraries. Some miRNAs were clearly expressed specifically at certain periods of development, indicating that these miRNAs may play important roles in ovary development. The miRNAs showed a wide range of expression patterns in the different developmental stages in this study, with 740 (31.06%), 433 (18.17%), 150 (6.30%), 628 (26.36%) and 431 (18.09%) miRNAs detected uniquely in the five libraries, respectively (Fig. 2). The neurula stage included 740 specifically expressed miRNAs, of which the most abundant were miR-518-3p, miR-2944-3p, miR-76-5p, and miR-1000-5p. The complete yolk sac absorption stage library included 433 specifically expressed miRNAs, the most abundant of which were miR-724-3p, miR-57-5p, miR-1612-3p, and miR-50-5p. A total of 150 miRNAs were specifically expressed in primordial gonads, with miR-1563-5p, miR-1420-3p, miR-6758-3p, and miR-1664-5p being the most abundant. Mir-1563-5p was extremely specifically expressed in primordial gonads, but with only a single count. Among the novel miRNAs, novel-m2571-5p, novel-m2117-3p, and novel-m2915-3p were highly specifically expressed in primordial gonads, with dozens of reads. A total of 628 miRNAs were specifically expressed in juvenile ovary, with the most abundant being miR-6984-5p, miR-8499-3p, miR-3177-3p, and miR-548-5p, while 431 miRNAs were specifically expressed in adult ovary.

Let-7, which is one of the most conserved miRNAs, has evolved into an miRNA family represented by eight members in Yellow River carp. Many other miRNA families were among the conserved Yellow River carp miRNAs, of which miR-100, miR-92, miR-124, miR-200, and miR-184, together with let-7, were the most highly conserved.

### Expression patterns of miRNAs at different ovary development stages in Yellow River carp

**Fig. 3:**
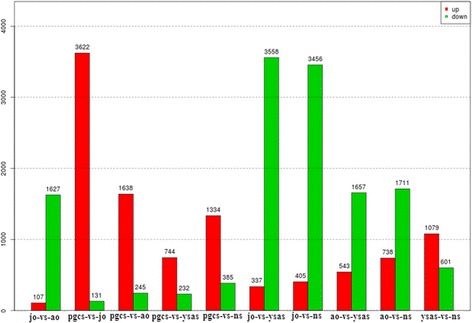
Differential expressions of miRNAs in Yellow River carp. Greater than 2-fold change while P < 0.05

We constructed a histogram of differentially expressed miRNAs in the five developmental stages using tags per million (TPM) normalized data (Fig. 3). The difference in miRNA expression was most significant between juvenile ovary and the other four stages. Compared with juvenile ovary, 3,456 miRNAs were down-regulated in the neurula stage, including miR-1 and miR-101a, while 405 miRNAs were up-regulated. A total of 3,558 miRNAs were down-regulated in the complete yolk sac absorption stage compared with juvenile ovary, including let-7, while 377 miRNAs were up-regulated. In primordial gonad, 3,622 miRNAs were up-regulated, including let-7, while 131 miRNAs were down-regulated, and 1,627 miRNAs were down-regulated in adult ovary, while 107 were up-regulated. The deep-sequencing approach allowed us to estimate the expression profiles of the miRNAs by calculating the sequence frequencies. The proportion of different categories of miRNAs reflects their functions in different developmental stages and the associated biological mechanisms.

**Fig. 4:**
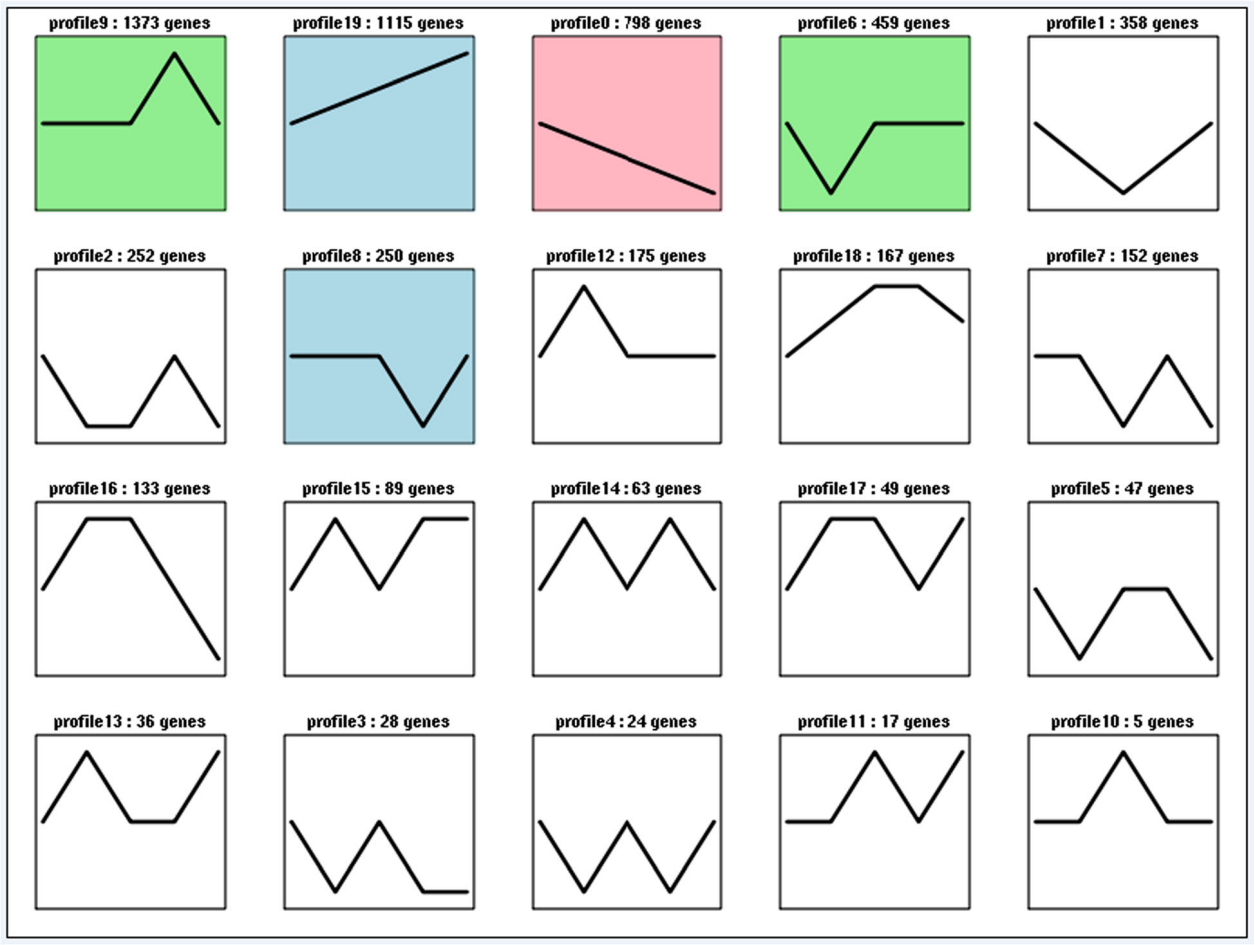
Trend analysis of miRNA expression profiles among different stages (abbreviations as in Fig. 1)

Trend analysis of miRNA expression among different developmental stages identified 20 different expression patterns (Fig. 4, Additional file 5), including 1,115 miRNAs that were up-regulated and 798 that were down-regulated during the process of ovarian differentiation (Fig. 4, profiles 19, 0). Expression of 1,373 miRNAs, such as mir-2779 and mir-9226-5p, were consistent between the neurula and yolk sac absorption stages, but increased from primordial gonad to juvenile ovary, and then decreased again in adult ovary (Fig. 4, profile 9). In contrast, 250 miRNAs showed the opposite expression pattern during ovary development, including miR-196a and miR-551 (Fig. 4, profile 8).

miRNAs targeting male-biased genes showed an upward trend. miR-148, miR-190, and miR-722 which were predicted to target *sox9* increased from the neurula stage to the primordial gonads, reached a peak in the primordial gonad and juvenile ovary stages, and then decreased in adult ovary. (Fig. 4, profile 18). *Gsdf* was the predicted target of ccr-miR-142-3p, ccr-miR-146a, and ccr-miR-214, which also increased from the neurula stage to primordial gonads, peaked in the primordial gonad and juvenile ovary stages, and then decreased in adult ovary (Fig. 4 profile 18). *Gsdf* was also the target of ccr-miR-22a, which showed increased expression from the neurula stage to adult ovary (Fig. 4, profile 19). The expression profiles of miR-200b and miR-201a, which were predicted to target *atm*, were also consistent with the above miRNAs with predicted male-biased target genes (Fig. 4, profile 18). In contrast, miRNAs targeting female-biased genes showed a downward trend. Expression levels of miR-203 and miR-203b-3p, which were predicted to target Smad4, decreased from the neurula stage to adult ovary (Fig. 4, profile 0).

The most abundant miRNAs in the present study were miR-430, miR-21, miR-22a, miR-181a, let-7a, miR-199-5p, miR-9-3p, miR-125b, miR-26a, miR-101a, miR-143, and miR-200a, which were not only co-expressed, but also significantly differentially expressed among the five different development stages in Yellow River carp. Expression of miR-430 decreased from the neurula stage to adult ovary (Fig. 4, profile 0). Let-7a was highly abundant and increased throughout the ovary development period (Fig. 4, profile 19), as did expression of miR-21 (Fig. 4, profile 19). These results suggest that these miRNAs may influence ovary development.

**Fig. 5:**
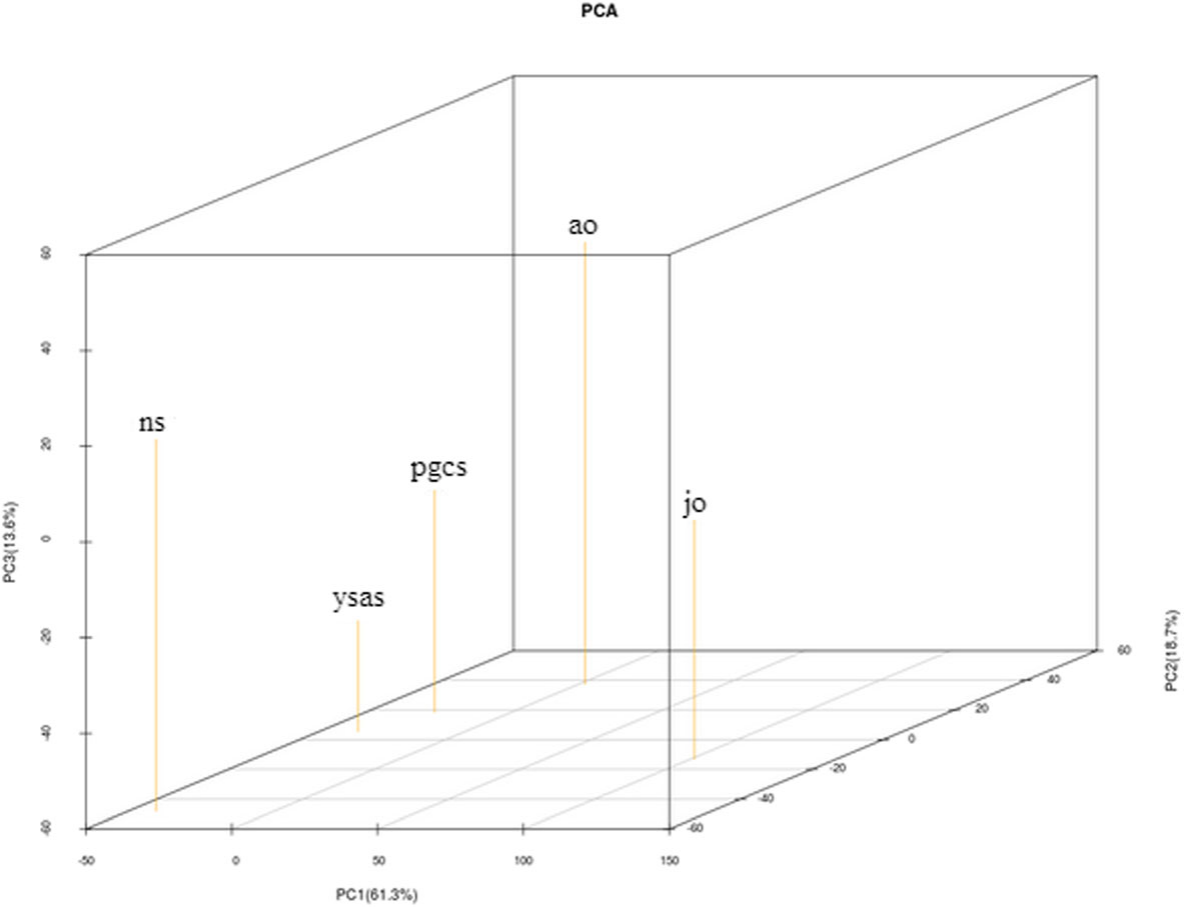
Three dimensional PCA map of the relationship of miRNA expression among different stages

We also analyzed the relationships of miRNA expression profiles among different developmental stages in the Yellow River carp (Fig. 5). Interestingly, the neurula and yolk sac absorption stages, primordial gonad, and adult ovary stages were relatively similar, but all were more distantly related to juvenile ovary (Fig. 5). This is probably because the ovary of adult fish has matured and reached the final stage of development, and it also indicates a new stage of development is about to begin. So, compared with juvenile ovary, the relevance between adult ovary and the other three early developmental stages are close.

### Prediction of target genes and signaling pathways analysis

We performed target-gene prediction based on the common carp (*C. carpio*) genome sequence (http://www.carpbase.org/) to identify the miRNAs involved in ovary development. A total of 26,569 putative target genes were predicted (Additional file 6). Kyoto Encyclopedia of Genes and Genomes (KEGG) functional annotation identified 239 annotated signaling pathways, including at least 10 pathways involved in reproductive biology, including transforming growth factor-β (TGF-β) signaling, Wnt signaling, oocyte meiosis, mitogen-activated protein kinase (MAPK) signaling, Notch signaling, p53 signaling, gonadotropin-releasing hormone (GnRH) signaling, RNA polymerase, steroid hormone biosynthesis, and metabolism of xenobiotics by cytochrome P450. Interestingly, the target genes of 494 miRNAs belonged to the MAPK signaling pathway, which plays an important part in virtually every step of spermatogenesis in the testis. The MAPK signaling pathway is also involved in the acrosome reaction in the female reproductive tract before fertilization of the ovum [11]. Wnt signaling is known to be involved in mammalian reproduction [38], and we detected 260 miRNA targets belonging to the Wnt signaling pathway, 161 belonging to the TGF-β signaling pathway, and 188 belonging to the GnRH signaling pathway. Given the important roles of steroid hormones in reproduction and sexual dimorphism in fish, we analyzed the relationships between miRNA and mRNA and the steroid hormone biosynthesis pathway, including *hsd11b* and *hsd3b*, which encode key enzymes in the steroid hormone biosynthesis pathway. *foxl2*, *stat1*, *sf1*, *dmrt1* and *gsdf* have been shown to be key factors in early ovary differentiation [39, 40]. We also analyzed *smad3*, *smad4*, *sox9*, and *atm*, which are also known to be responsible for gonad differentiation, and found that these genes were predicted targets of many miRNAs, which could thus negatively regulate these pathway genes.

*Foxl2* was a predicted target of miR-132b, miR-135c, and miR-138. miR-138 expression was down-regulated during all five ovary development stages, miR-132b was down-regulated from the primordial gonad, and miR-135c was down-regulated from the yolk sac absorption stage. MiR-132a and miR-181, which targeted *dmrt2*, were up-regulated, while miR-148 and miR-193a, which were predicted to target *smad3*, were also up-regulated, and miR-138 targeting *amh* was down-regulated. *Gsdf* was the predicted target of miR-132a, miR-146a, miR-210, miR-214, miR-22, and miR-22b; *Hsd11b* was predicted to target miR-1020-3p and miR-1187-3p; *Hsd3b* was the predicted target of miR-133-3p and miR-191-5p; *Stat1* was predicted to be the target of miR-101a and miR-181a; *Sf1* of miR-101b and miR-144; *Sox9* of miR-15b and miR-16a; and *atm* was predicted to be the target of miR-132a and miR-181. These results illustrate the possible roles of the differentially expressed miRNAs in ovary differentiation and development, though further studies are needed to clarify their functions.

A total of 150 miRNAs were specifically expressed in primordial gonads, of which novel-m2571-5p miRNA had the highest expression. This miRNA was also predicted to be involved in many reproductive biology pathways, including steroid metabolic processes, TGF-β receptor signaling, Wnt signaling, and cell differentiation. Its predicted target genes included *cyp51a1*, *hsd3*, *smad4*, *lemd3*, *zranb1*, *tbx6*, *grk6*, *ccna1*, and *prosapip1*, of which some, such as *cyp51a1* and *hsd3*, were gonad development-related genes. Many other miRNAs specifically expressed in primordial gonad were also predicted to target genes associated with reproductive processes. MiR-1563-5p was predicted to target *bcl9* and *notch2*, which belong to the TGF-β signaling and Notch signaling pathways, respectively, and miR-1420-3p was predicted to target *pdk1* and *inhibin beta A chain*, which are related to TGF-β signaling and female gonad development, respectively. Although the predicted target genes need to be validated experimentally, these results illustrate some of the possible roles of the miRNAs specifically expressed in ovary reproductive processes. The miRNAs specifically expressed in primordial gonad may thus be involved in ovary development, and their importance in ovarian differentiation mechanisms need to be clarified.

### Nucleotide bias of miRNAs in Yellow River carp ovary

**Fig. 6:**
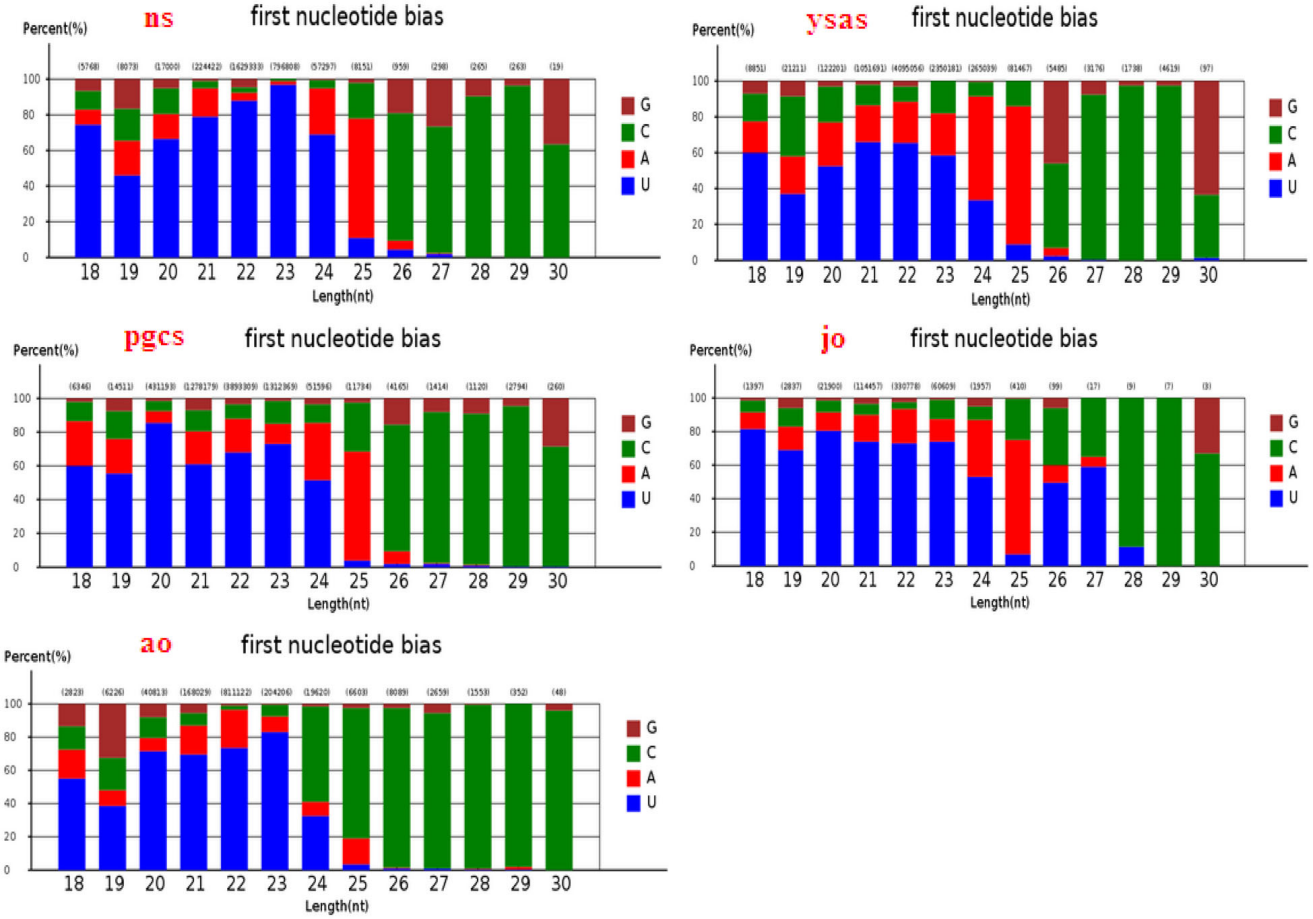
First-nucleotide bias in different length tags in conserved miRNAs of Yellow River carp

**Fig. 7:**
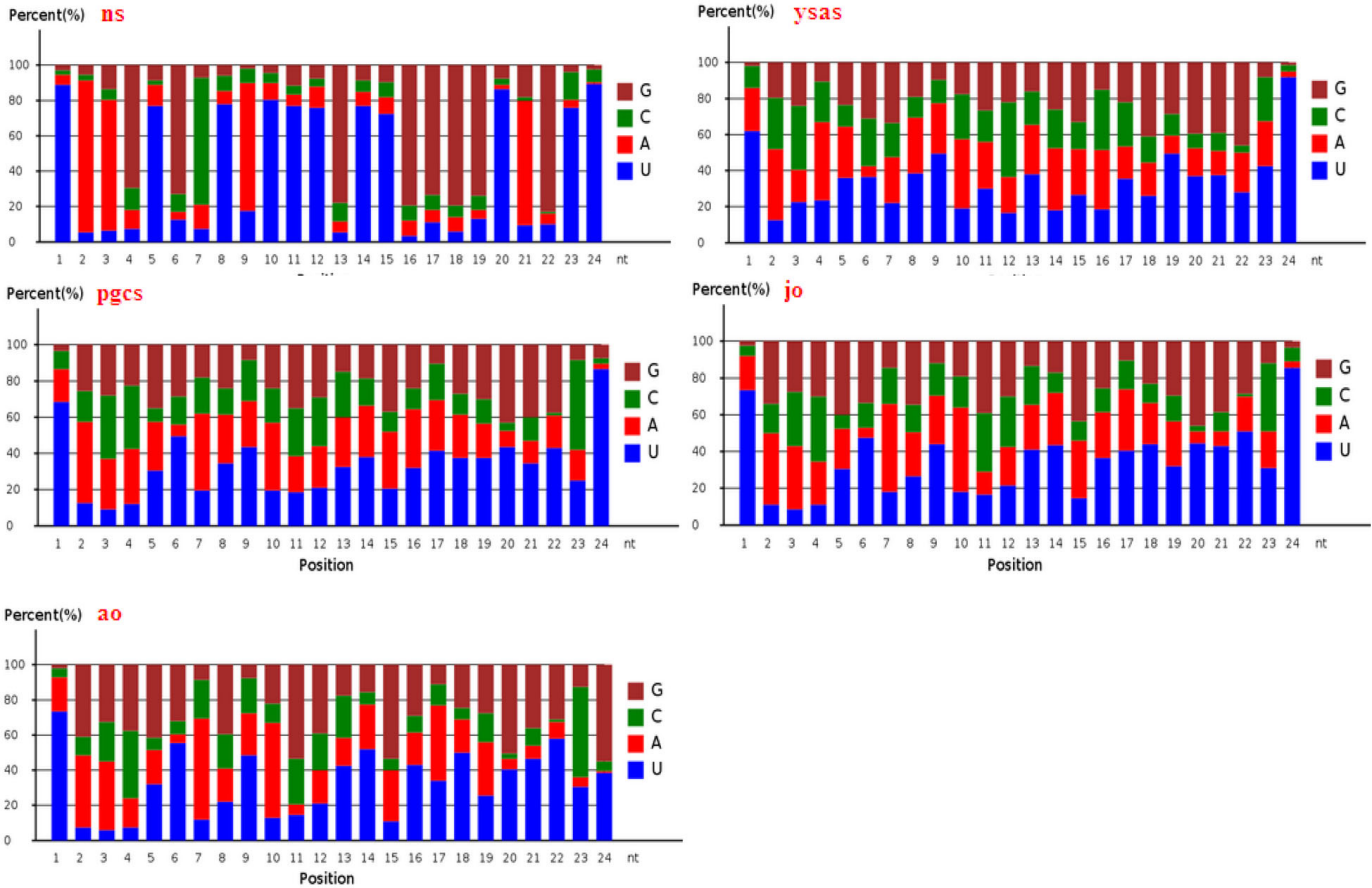
Nucleotide bias at each position in conserved miRNAs of Yellow River carp

**Fig. 8:**
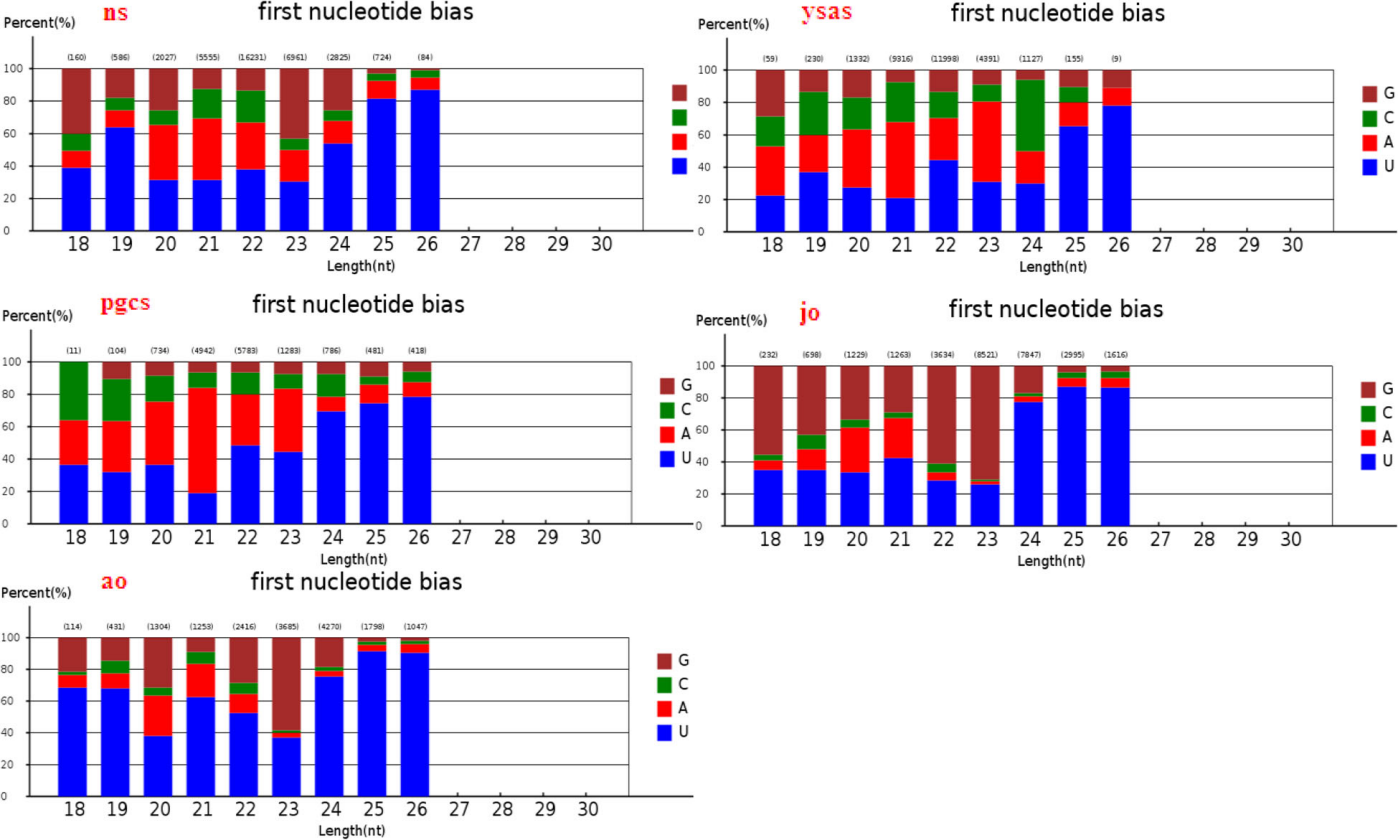
First-nucleotide bias in different length tags in novel miRNAs of Yellow River carp

We investigated the nucleotide bias of miRNAs in carp ovary development. U was the most common nucleotide at the 5’ end of the conserved miRNAs, accounting for 72% of the 25-nt miRNAs in the neurula stage, 79% of the 24-nt miRNAs in the complete yolk sac absorption stage, 85% of the 20-nt miRNAs in primordial gonads, 85% of the 22-nt miRNAs in juvenile ovary, and 85% of the 23-nt miRNAs in adult ovary (Fig. 6). These results were consistent with other studies that also found U to be the most common base at the extreme 5’ end [41–43]. In the case of novel miRNAs, U was the most common 5’ base in 87% of the 26-nt novel miRNAs in the neurula stage, 78% of the 26-nt miRNAs in the complete yolk sac absorption stage, 78% of the 26-nt miRNAs in primordial gonads, 87% of the 25-nt miRNAs in juvenile ovary, and 91% of the 25-nt miRNAs in adult ovary (Fig. 7). Interestingly, U was the dominant first nucleotide of the novel 19-, 22-, 23-, 24-, 25-, and 26-nt miRNAs in the neurula stage, yolk sac absorption stage, and primordial gonads, while A was the dominant first nucleotide of the 20- and 21-nt novel miRNAs. U was the dominant first nucleotide of all the novel 18–26-nt miRNAs in juvenile and adult ovaries (Fig. 8). The phenomenon of nucleotide bias might be related to the mechanisms of miRNA action, such as binding to the target gene.

**Fig. 9:**
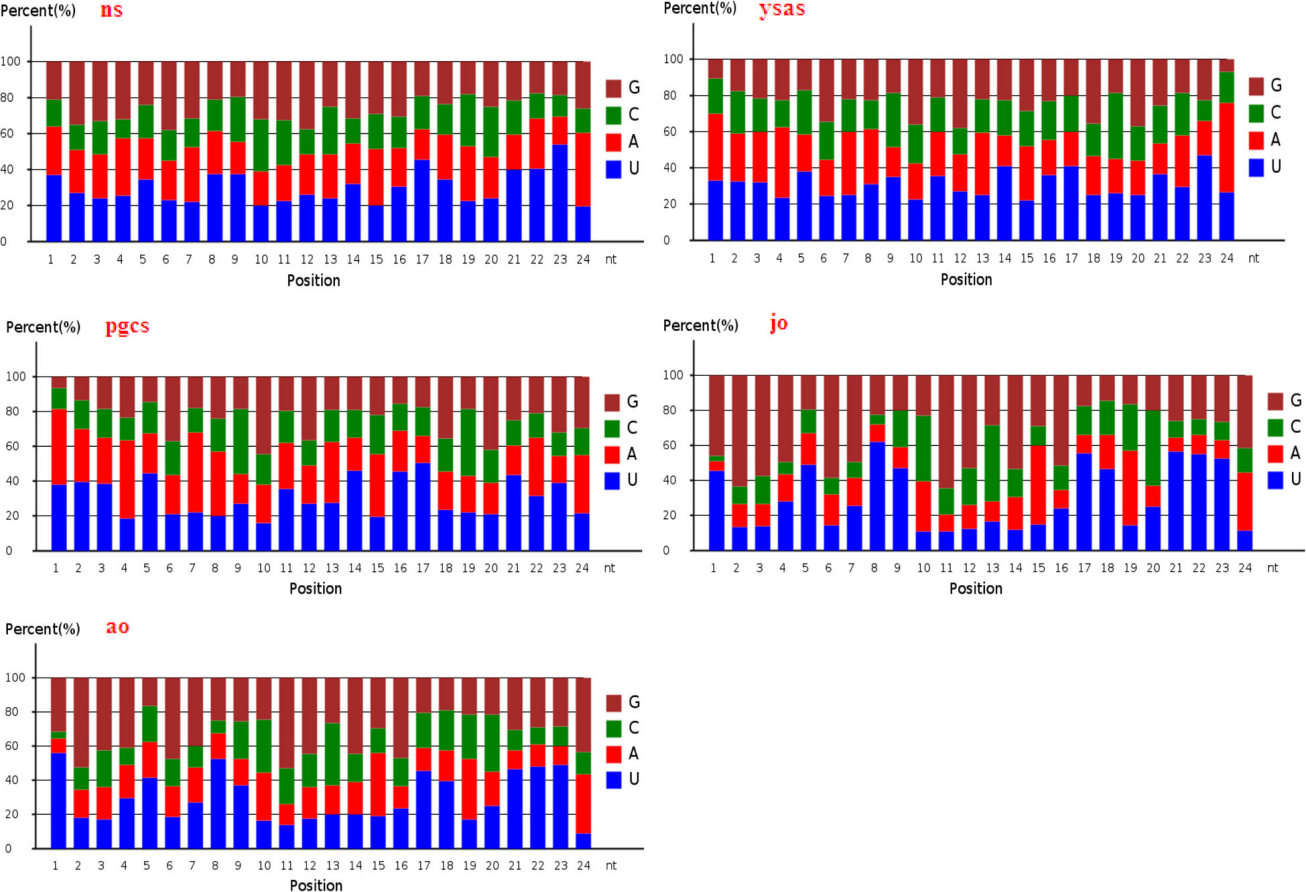
Nucleotide bias at each position in novel miRNAs of Yellow River carp

Base composition is a fundamental feature of miRNA sequences. It influences their physiochemical and biochemical properties, as evidenced by the effects of secondary structure on the activity of enzymes and miRNAs [44–47]. Nucleotide bias analysis at each position showed that U and A were mainly located at the beginning and end of reads of conserved miRNAs and novel miRNAs (Figs. 7 and 9), suggesting that AU base pairing may affect miRNA secondary structure or target recognition [47].

### Validation of miRNAs by quantitative polymerase chain reaction

**Fig. 10:**
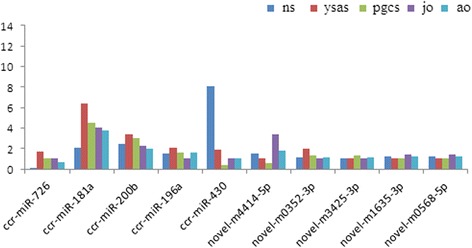
Real-time quantitative PCR validates expressions of 10 randomly selected miRNAs. The amount of expression was normalized to the level of U6 snRNA

We verified the results of Solexa sequencing by quantitative real-time polymerase chain reaction (qRT-PCR) analysis of 10 randomly selected miRNAs, including five conserved miRNAs (ccr-miR-726, ccr-miR-181a, ccr-miR-200b, ccr-miR-196a, ccr-miR-430) and five novel miRNAs (novel-m4414-5p, novel-m0352-3p, novel-m3425-3p, novel-m1635-5p, novel-m0568-5p). The relative expression levels of all 10 miRNAs were consistent with the sequencing data (Fig. 10), indicating the reliability of the miRNA expression and correlation analyses based on small-RNA sequencing.

## Discussion

Increasing evidence suggests that miRNAs act as important regulators of reproduction via controlling the expression of a vast number of genes [48–50]. However, their exact functions in lower vertebrates, such as fish, remain poorly understood. We performed an integrated analysis of deep-sequenced miRNA throughout ovary development (primordial gonad, juvenile ovary and adult ovary) and two embryo stages (neurula stage and complete yolk sac absorption stage) in female Yellow River carp to identify miRNAs with important roles in female gonad development. Nervous system differentiation starts during the neurula stage, and the HPG axis plays an important role in fish gonad differentiation. Complete yolk sac absorption represents a key period for larval growth, during which metabolic exchange with the external environment is initiated, marked by the start of expression of a large number of genes [32, 33]. Primordial germ cell formation is a crucial stage of ovary differentiation, and comparative analysis of miRNA expression profiles between this and the other four stages may reveal miRNAs with important roles in ovary differentiation.

The miRNA libraries constructed from the neurula stage, yolk sac absorption stage, and primordial gonads displayed similar read-length distributions of 22 and 23 nt, followed by 21 nt, representing the typical size of Dicer-derived products. However, the length distributions of miRNAs in mouse ovary and tilapia gonads at an early stage of ovary differentiation were 21 and 22 nt [8, 51], which differed from the present results. The length distribution increased in juvenile and adult ovary libraries, with an increase in 25–28-nt miRNAs in mature ovary, possibly indicating the abundant expression of piRNAs. This phenomenon was also observed in a previous study of gonadal miRNAs in tilapia and zebrafish [52, 53]. The above results indicated that miRNA length increased with increasing ovary differentiation. Furthermore, increasing tissue differentiation was associated with increased expression of miRNAs related to the tissue function, such as piRNAs, accounting for the difference in average length in mature ovaries.

The most abundant miRNAs in primordial gonads were miR-430, miR-21, let-7a, miR-199-5p, miR-9-3p, miR-22a, miR-125b, miR-26a, miR-181a, miR-101a, miR-143, and miR-200a with >10,000 reads each. These miRNAs were also highly expressed in other developmental periods. Some miRNAs showed similar high-expression patterns in Yellow River carp, bighead carp, and silver carp [54]. MiR-122, let-7, miR-192, miR-21, miR-499, miR-146, miR-101, miR-128, miR-26, and miR-124 were highly expressed in adult bighead carp and silver carp [54]. A previous study indicated that miR-21 played a role in preventing apoptosis in periovulatory granulosa cells as they transit to luteal cells [55], while miR-21 expression in cattle was also significantly increased in ovary compared with testis, suggesting that miR-21 may play a regulatory role in female physiology [12]. Has-miR-21 was also up-regulated by ovarian steroids in mouse granulosa cells and human endometrial stromal cells or glandular epithelial cells [56, 57]. In this study, miR-21 was abundant in all five stages, but was obviously up-regulated in the primordial gonad. The predicted target genes of miR-21 included genes in the MAPK, B-cell receptor, TGF-β, and apoptotic pathways, suggesting that it may play crucial roles in ovary development, gonadal differentiation [58], and endocrine regulation [11, 59]. The miR-430 family is known to be involved in embryonic morphogenesis and clearance of maternal mRNAs [60–63], and miR-430 was significantly expressed in all five tested stages of Yellow River carp development. The miR-430 family was similarly highly expressed during early zebrafish development [61]. MiR-430 has been shown to target chemokine signaling to ensure accurate migration of primordial germ cells [64]. Let-7 was another highly-expressed miRNA family in all five stages in the current study. The let-7 family was first discovered and characterized in *Caenorhabditis elegans*, and plays an important role in regulating late developmental events by down regulating *lin-41* and possibly other genes [65]. Let-7 was the third most highly expressed miRNA in all five stages in Yellow River carp. MiR-143 was highly expressed in Yellow River carp, and was also shown to be a dominant miRNA in ovaries in cattle, pigs, and yellow catfish [10, 23]. The miR-181a family is abundantly expressed in the gonads of tilapia [18], mice [66], and humans [67]. Overall, these results suggest that miR-430, miR-21, let-7, miR-181a, and miR-143 may play important roles in ovary differentiation and development in Yellow River carp.

Some abundantly expressed miRNAs are highly conserved in evolution and are considered to be housekeeping miRNAs involved in the maintenance of basic cellular activities. In contrast, miRNAs such as miR-1563-5p, miR-1420-3p, miR-6758-3p, and miR-1664-5p have important regulatory functions in cell differentiation and were specifically expressed in primordial gonads. These miRNAs were involved in ovary differentiation in vertebrates, and may be considered as ‘luxury’ miRNAs. Notably, most of the novel miRNAs identified in this study were weakly expressed (tens to hundreds of reads), in accordance with the results for *Drosophila*, and bighead and silver carp [54, 68].

Differentially expressed miRNAs showed a variety of expression patterns at different development stages. Among the 20 different expression patterns, two patterns are particularly worthy of attention, involving miRNAs with expression levels that either increased or decreased significantly from primordial gonads to mature ovary. These miRNAs may be direct regulators of ovary differentiation. For example, mir-2779 and mir-9226-5p increased from primordial gonads to juvenile ovary and then decreased in mature ovary, while miR-196a and miR-551 decreased over the same period and then increased in mature ovary. Compared with other stages, expression of specific miRNAs was reduced in primordial gonads, possibly because of the presence of fewer cell types and the lower degree of differentiation at this stage. Stage-specifically expressed miRNAs may play important roles at particular developmental stages, and further studies are needed to characterize the functions and target genes of these miRNAs in primordial gonads. Let-7 was the most significantly differentially expressed miRNA during the process of ovary development in the current study, with a significant increase from the neurula stage to juvenile ovary. This suggests that these miRNAs may have a vital function in the timing of ovary developmental. Comparison of miRNA expression profiles among the different developmental stages of Yellow River carp indicated relatively close relationships among the neurula stage, yolk sac absorption stage, primordial gonad, and adult ovary, all of which were less similar to the profile in juvenile ovary. This may be due to the status and role of different developmental stages during ovary development, as well as the specificity of miRNA expression.

MiR-27 may be involved in the regulation of cell proliferation and differentiation in vertebrates, but has not been found in invertebrates. It has been suggested that miR-27 may have originated in fish [54]. Five members of the miR-27 family have been identified to date, including three in pufferfish (*Fugu rubripes* and *Tetraodon nigroviridis*), four (hno-miR-27a, hon-miR-27b, hno-miR-27d, hno-miR-27e) in bighead and silver carp [54], and five (dre-miR-27a, dre-miR-27b, dre-miR-27c, dre-miR-27d, dre-miR-27e) in zebrafish [53]. We found five members of the miR-27 family in Yellow River carp, including ccr-miR-27a, ccr-miR-27c-3p, ccr-miR-27c-5p, ccr-miR-27d, miR-27-5p, and miR-27-3p. These studies indicate that different miRNA family members occur in different species of the same lineage. In addition, up-regulation of miR-27 members suggests a possible role of this family during ovary development.

Target-gene prediction showed that many of the target genes identified were key factors involved in sex differentiation. Among these predicted genes, *sox9*, *dmrt1*, and *gsdf* have been identified as sex-determining genes in fish species [69, 70], and were identified as the targets of some of the miRNAs in our study (*hsd3*/novel-m2571-5p; *gsdf*/ccr-miR-146a, ccr-miR-214, ccr-miR-22a; *foxl2*/miR-132b, miR-135c, miR-138). *Hsd11b* and *hsd3b* encode key enzymes in the steroid hormone biosynthesis pathway. These genes may participate in steroid hormone synthesis, gonadal function, and mechanisms of sex-differentiation, and may play a vital role in developmental timing. However, further studies are needed to confirm these interactions and functions.

## Conclusions

This study provides the first report of the differential miRNA expression profiles in five important ovary development stages in Yellow River carp, and provides initial data regarding the potential involvement of miRNAs in ovary development. miRNAs are widely involved in ovary differentiation, including housekeeping miRNAs with high expression levels at all stages, and ‘luxury’ miRNAs with higher levels of expression at specific stages, including 150 and 628 miRNAs specifically expressed in primordial gonads and juvenile ovary, respectively. These specifically expressed miRNAs provide the basis for further studies to clarify the role of miRNAs in the early stage of ovarian differentiation. Special attention should be paid to miRNAs with different expression patterns in primordial gonads and juvenile ovary, given that the target genes of these miRNAs include genes known to be involved in sex determination and early gonadal differentiation in fish. Understanding the mechanisms whereby specifically expressed miRNAs interact with their target genes is crucial for revealing the mechanisms of ovary differentiation. KEGG pathway analysis identified numerous signaling pathways involved in ovary development, including the TGF-β, Wnt, MAPK, and Notch signaling pathways, indicating the need to study the mechanisms of ovary differentiation in fish from a wider perspective.

## Methods

### Fish samples

All investigations in this study were performed according to the Animal Experimental Guidelines of the Ethical Committee of the University of China. The Yellow River carp used in this study were obtained from the aquaculture base of Henan Normal University. Embryos were obtained by natural spawning and cultured in embryo medium following standard procedures. The test samples consisted of ovaries from three different developmental stages and two embryonic stages. Samples of primordial gonads were collected from larvae at 45 days post-hatching (dph), based on the results of our previous studies. The original reproductive gland was dissected under a microscope, and samples from 50 fish were mixed after confirmation by histological section. Samples of juvenile ovary were collected from 30 of 80 dph female fish, and stage II ovaries were confirmed by histological sections. Adult ovary samples were collected from 10 of 3 aged sexually mature female fish, and the ovaries were confirmed as stage V. Whole neurula stage embryos were collected at 13 h post-fertilization, and complete yolk sac absorption stage embryos were collected as whole larva at 2 dph. The tissues were immediately frozen in liquid nitrogen and stored at −80 °C for further use.

### RNA isolation

Total RNA was extracted from each sample separately using TRIzol reagent (Invitrogen, Carlsbad, CA, USA) following the manufacturer’s protocol. The quantity and purity of total RNA were checked using the Agilent 2100 Bioanalyzer system (Santa Clara, CA, USA) and denaturing gel electrophoresis, and the samples were then stored at −80 °C.

### Small-RNA library construction and sequencing

We generated small-RNA libraries from the five samples from Yellow River carp using the mirVana^TM^ mircoRNA Isolation Kit (Ambion, USA), according to Guideline. Five biological replicates were prepared for each sample of three different ovary developmental stages, with each biological replicate comprising RNA extracted from 10 to 50 fish. Five biological replicates were prepared for each sample for both embryo stages, with each biological replicate comprising RNA extracted from 30 to 50 embryos or larvae. A total of 25 small-RNA libraries were prepared from the five samples, with five biological replicates for each sample. Total RNA was ligated with 3’ and 5’ RNA adaptors, and fragments with adaptors on both ends were enriched by PCR after reverse transcription. The subsequent cDNAs were purified and enriched by 6% denaturing polyacrylamide gel electrophoresis to isolate the expected size fractions and eliminate unincorporated primers, primer dimer products, and dimerized adaptors. Finally, the five RNA libraries were sequenced using an Illumina/Solexa Genome Analyzer at Guangzhou Genedenovo Biotech Company (Guangzhou, China).

### Analysis of sequencing data

The raw sequence data were filtered to remove low-quality reads and adaptor sequences. After adaptor trimming, reads of 16–35 nt in length were kept for further bioinformatic analysis. The remaining reads were mapped to the *C. carpio* genome with a tolerance of zero mismatches in the seed sequence using Bowtie (version 1.1.0). Sequences mapping to the genome were remained for further analysis. The reads mapped to the *C. carpio* genome were subsequently analyzed to annotate rRNA, tRNA, snRNA, snoRNA, and non-coding RNA sequences by blasting against the Rfam database (11.0, http://rfam.xfam.org) and GenBank data base (http://www.blast.nvbi.nlm.nih.gov/). The remaining sequences were identified as the conserved miRNAs in carp by blasting against miRBase 21.0 allowing no more than two mismatches. Existing carp miRNA referred to *C. carpio* miRNA included in the miRBase with no base mismatch. The sequences that did not match existing or conserved miRNAs were used to identify potentially novel miRNA candidates [71, 72]. These were identified by folding the flanking genome sequence of unique small RNAs using MIREAP (https://sourceforge.net/projects/mireap/). The enrichment degree of each miRNA was identified by counting the number of reads in each sample. To determine differentially expressed miRNAs among the five libraries, the frequency of miRNA counts was normalized as transcripts permillion (TPM). The TPM was calculated as follows: normalized expression, TPM = (actual miRNA count/number of total clean reads) × 1,000,000. Only the miRNA with more than 2-fold change in the two compare samples were considered significantly differentially (*P*-value (<0.05)) [73] expressed miRNAs. A positive value indicated up-regulation of a miRNA, while a negative value indicated down-regulation.

Target genes of miRNAs were predicted using RNAhybrid(v2.1.2) + svm light(v6.01), miRanda(v3.3a) and Targetscan software. The overlap of the predicted results from the three programs was considered to represent the final result of predicted target mRNAs. Pathway analyses of the predicted target mRNAs was performed using the KEGG pathway database (http://www.genome.jp/kegg/pathway.html) [74].

### Quantitative PCR

The expression profiles of 10 randomly selected miRNAs, including five conserved and five novel miRNAs, were investigated by qRT-PCR to validate their expression changes. Total RNA (500 ng) was converted to cDNA using miScript reverse transcriptase mix (Qiagen, Valencia, CA, USA) according to the manufacturer’s instructions. qRT-PCR was carried out using an Applied Biosystems 7300 Real-Time PCR System according to the standard protocol. The cDNA samples were diluted to 1/150 and 5 μL were used for each real-time PCR reaction. The 20-μL PCR reaction mixture included 10 μL SYBR Premix Taq (2×), 0.4 μL miRNA-specific forward primers (10 μM), 0.4 μL miScript universal primer (10 μM), and 1 μL PCR template (cDNA). The reactions consisted of 50 °C for 2 min, followed by 40 cycles of 95 °C for 2 min, 95 °C for 15 s, and 60 °C for 30 s. Melting curve analyses were performed following amplification and standard curves for endogenous and all miRNAs were constructed using serial dilutions of a pooled cDNA sample. The respective standard curves were used to determine the quantity of the selected miRNAs and reference genes. All primers used in the qPCR experiments are shown in Additional file 7: Table S5. Relative miRNA expression levels were calculated using the 2-ΔΔCt method after the threshold cycle (Ct). Each sample was run in triplicate. snRNA U6 was used as an endogenous control for real-time PCR of miRNAs.

## Additional files

Additional file 1: Table S7. Brief summary of the samples and sequencing data of Yellow River carp. (DOCX 16 kb)

Additional file 2: Conserved miRNAs identified from the five development stages of Yellow River carp. (XLS 159 kb)

Additional file 3: Existing carp miRNAs predicted from the five development stages of Yellow River carp. (XLS 16 kb)

Additional file 4: Novel miRNAs predicted from the five development stages of Yellow River carp. (XLS 541 kb)

Additional file 5: Table S6. Trend analysis of differentially expressed miRNAs in the five development stages of Yellow River carp. (XLS 499 kb)

Additional file 6: Predicted target genes of differentially expressed miRNAs and target genes associated with significantly enriched GO terms in the five development stages of Yellow River carp. (XLS 108197 kb)

Additional file 7: Table S5. Primers used to validate the 10 selected miRNAs. (DOCX 16 kb)

## Abbreviations

*atm*: Ataxia telanglectasia mutated
*dmrt1*: Doublesex- and mab-3-related transcription factor-1
*foxl2*: Forkhead box L2
*gsdf*: Gonadal somatic cell derived factor
*hsd11b*: 11- hydroxysteroid dehydrogenase
*hsd3b*: 3 β- and steroid δ-isomerase 3
*sf1*: Steroidogenic factor-1
*smad3*: Mothers against decapentaplegic 3-like protein
*smad4*: Mothers against decapentaplegic 4-like protein
*sox9*: SRY-like HMG-box9
*stat1*: Signal transducer and activator of transcription 1
