# Supplementary material for: Identification and profiling of Cyprinus carpio microRNAs during ovary differentiation by deep sequencing
Source: BMC Genomics. 2017 Apr 28;18:333. doi: 10.1186/s12864-017-3701-y (PMC5410099; doi:10.1186/s12864-017-3701-y)
Supplement: Supplementary file 1 — Brief summary of the samples and sequencing data of Yellow River carp. (DOCX 16 kb) [file 12864_2017_3701_MOESM1_ESM.docx]

**Additional file 1: Table S7** Read statistics of the obtained small RNAs

| Samples Total reads Clean reads |
| --- |

|  |
| --- |

Neurula stage 16247107 15122610

Yolk sac complete absorption stage 15228538 14250458

Primordial gonad 16968093 15765913

Juvenile ovary 15722656 15157370

Adult ovary 14604487 13750335

|  |
| --- |
