# Supplementary material for: Identification and profiling of Cyprinus carpio microRNAs during ovary differentiation by deep sequencing
Source: BMC Genomics. 2017 Apr 28;18:333. doi: 10.1186/s12864-017-3701-y (PMC5410099; doi:10.1186/s12864-017-3701-y)
Supplement: Supplementary file 7 — Primers used to validate the 10 selected miRNAs. (DOCX 16 kb) [file 12864_2017_3701_MOESM7_ESM.docx]

**Additional file 7: Table S5** Primers for decting expression of miRNAs in carp

| Name | Sequence |
| --- | --- |
| miR-196a  miR-430  miR-181a  miR-200b  miR-726  novel-m4414-5p  novel-m0352-3p | TAGGTAGTTTCATGTTGTTGGG  TAATACTGTCTGGTAATGCCGT  AACATTCAACGCTGTCGGTGA TAATACTGCCTGGTAATGATGA  TTCACTACTAGCAGAACTCGG GCTAATACTGTCTGGTAATGCC  ATTATGAACATCGATCTTGCGT |
| novel-m3425-3p  novel-m1635-5p  novel-m0518-3p  U6-F | ATTATGAATGTTGATATTGCGT  ATATCCCGGACGAGCCCCCA  TGCAACATGAGTAGACGCTCGT  CTCGCTTCGGCAGCACA |
| U6-R | AACGCTTCACGAATTTGCGT |
|  |  |
